# Clipping versus coiling for the treatment of oculomotor nerve palsy induced by posterior communicating artery aneurysms: A comparison of effectiveness

**DOI:** 10.1002/brb3.2263

**Published:** 2021-06-21

**Authors:** Zidong Wang, Xiaokui Kang, Qingdong Wang

**Affiliations:** ^1^ Department of Neurosurgery Liaocheng People's Hospital Liaocheng Shandong P.R. China; ^2^ Department of Neurology Liaocheng People's Hospital Liaocheng Shandong P.R. China

**Keywords:** clip, coil, oculomotor nerve palsy, posterior communicating artery aneurysms

## Abstract

**Background:**

A long debate has been going on in the clinical effectiveness to determine whether surgical clipping or coiling more favorable for oculomotor nerve palsy (ONP) caused by PcomAA. We aimed to perform a study, focusing on the effectiveness of ONP induced by PcomAA after treatment of surgical clipping and endovascular coiling.

**Method:**

Potential studies were searched on PubMed, EMBASE, Web of Science, and Cochrane Library from database inception to February 2021, and STATA version 12.0 was exerted to process the pooled data.

**Results:**

A total of 16 articles are included in the study, hailing from the United States, South Korea, the United Kingdom, France, Germany, Korea, China, Japan, Britain, and Singapore. The results showed that the clipping group was related to a higher incidence of complete ONP recovery at follow‐up (OR = 5.808, 95% CI 2.87 to 11.76, *p* < 0.001), the lower rates of partial ONP recovery (OR = 0.264, 95% CI 0.173 to 0.402, *p* < .001) and no improvement of ONP at follow‐up (RD = –0.149, 95% CI –0.247 to –0.051, *p* = .003). In the subgroup of complete ONP recovery based on the condition of patients, clipping was associated with a higher incidence of complete ONP recovery in patients with the incomplete initial ONP (OR = 3.579, *p* = .020) and ruptured aneurysm (OR = 5.38, *p* = .020). Regarding the subgroup of complete ONP recovery based on the quality of studies, similar results also appeared.

**Conclusion:**

Surgical clipping was more favorable to the recovery from ONP caused by PcomAA endovascular coiling due to a higher rate of recovery and recovery degree of ONP. Besides that, more evidence‐based performance is necessary to supplement this opinion.

## Graphical abstract

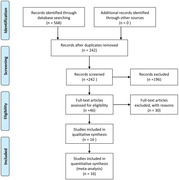

**Abbreviations**
CIs: confidence intervals
non‐RCTs: nonrandomized controlled trials
NOS: Newcastle–Ottawa scale
ONP: oculomotor nerve palsy
ORs: odds ratios
PcomAA: posterior communicating artery aneurysm
PRISMA: Preferred Reporting Items for Systematic Reviews and Meta‐Analyses
RDs: rate differences

## 1 INTRODUCTION

Posterior communicating artery aneurysm (PcomAA) ranked the second most common intracranial aneurysm, accounting for 25% of all cerebral aneurysms (Golshani et al., 2010). Oculomotor nerve palsy (ONP) was a pretty common complication of PcomAA; it was reported that ONP occurred in more than one‐third of patients with PcomAA because of the adjacent anatomical relationships, pulsating effect from aneurysms, and direct nerve stimulation caused by subarachnoid hemorrhage (Giombini et al., 1991; Kh et al., 2010; Patel et al., 2014 ). It could be complete or incomplete and be related to ophthalmoplegia, diplopia, and ptosis (Gaberel et al., 2016). Recovery and the degree of recovery of ONP caused by PcomAA often showed a profound effect on the patient's physical health and quality of life. Surgical clipping and coiling were the two most common therapy for ONP caused by PcomAA. Clipping was a recommended treatment for ONP induced by PcomAA, which was a straightforward treatment by placing a metal clip at the lesion of the vessel and associated with good results in the majority of patients by achieving immediately relieves mass effect (Golshani et al., 2010; Lq & Qx, 2020 ). As the treatment of coiling, the spring‐shaped coil was placed at the lesion of aneurysms by adopting a catheter via the groin artery (Lq & Qx, 2020 ). The endovascular treatment could remove aneurysmal pulsation and tended to promote the atrophy of aneurysm over time (Mc et al., 2008; Panagiotopoulos et al., 2011; Sh et al., 2010; Sz et al., 2010 ). Therefore, given that the controversy between the endovascular coiling and surgical clipping for ONP induced by PcomAA remained unresolved, we aimed to perform a study, focusing on the effectiveness of ONP induced by PcomAA after treatment of surgical clipping and endovascular coiling.

## 2 METHODS

This review works fully followed the checklist of PRISMA (Preferred Reporting Items for Systematic Reviews and Meta‐Analyses) (Wq et al., 2019).

### 2.1 Ethical review

All analyses were conducted according to available published studies, thus no ethical approval or patient consent was required.

### 2.2 Literature search

The analysis of all literature that compared surgical clipping with endovascular coiling for patients with ONP induced by PcomAA was searched by two authors through four medical publication databases, namely PubMed (up to October 2020; updated February 2021), Cochrane Central Register of Controlled Trials (up to October 2020; updated February 2021), EMBASE (up to October 2020; updated February 2021), and Web of Science (up to October 2020; updated February 2021). The search strategy adopted both keywords and the MeSH term searches about “aneurysm,” “clip,” “oculomotor nerve palsy,” and “coiling,” which were also in combination with Boolean logic. Two authors conduct an initial search independently, and controversy was discussed and deal with by the third author. The search was limited to English‐language articles, and a manual search of the reference lists of the included studies was performed as well.

### 2.3 Selection criteria

Publications were selected on the basis of the PICOS criteria (population, intervention, comparison, outcome, and study design) as follows: (I) P: limited to participants with ONP induced by PcomAA; (II) I: adopted endovascular coiling or surgical clipping as the intervention; (III) C: the comparative studies between coiling and surgical clipping; (IV) O: Included one or more endpoints mentioned above; (V) S: randomized controlled trial (RCT) or non‐RCT.

Conversely, the exclusion criteria were exhibited below: (I) studies without comparison between coiling and surgical clipping; (II) animal model and basic science studies; (III) conference, case reports or/and series, editorials, letters to the editor, systematic reviews.

### 2.4 Data extraction

Two authors independently extracted the data, and a third author could join the process of in this part in case of discrepancy. The basic demographic information (the mean age, gender, study design, the number of patients) was extracted based on a preplanned form.

#### 2.4.1 The extracted outcomes included

Primary outcome measures: complete ONP recovery at follow‐up, the subgroup of patients with good or nongood control of confounding factors, the subgroup of study based on NOS scores, the subgroup of patients with complete or incomplete initial ONP, and the subgroup of patients with the ruptured or unruptured aneurysm.

Secondary outcome measures: partial ONP recovery, no ONP improvement at follow‐up, and postoperative complications.

### 2.5 Statistical analysis

Our statistical analyses were conducted using STATA 12.0 software (Stata Corporation, College Station, TX, USA). The pooled odds ratios (ORs) or risk differences (RDs) and their corresponding 95% confidence intervals (CIs) were assessed using a fixed‐effect model or random‐effect model. Statistical heterogeneity was assessed using Cochran *Q* test and *I* ^2^ statistic, to evaluate the degree of inconsistency among all included studies. The *I* ^2^ > 50% and < 50% were considered to have significant heterogeneity or not, respectively. The random‐effect model was adopted for variables with values of *I* ^2 ^> 50%. Otherwise, the fixed‐effect model was used. Two‐sided *p* values < .05 were regarded to have statistical significance.

## 3 RESULTS

### 3.1 Search results

**FIGURE 1:**
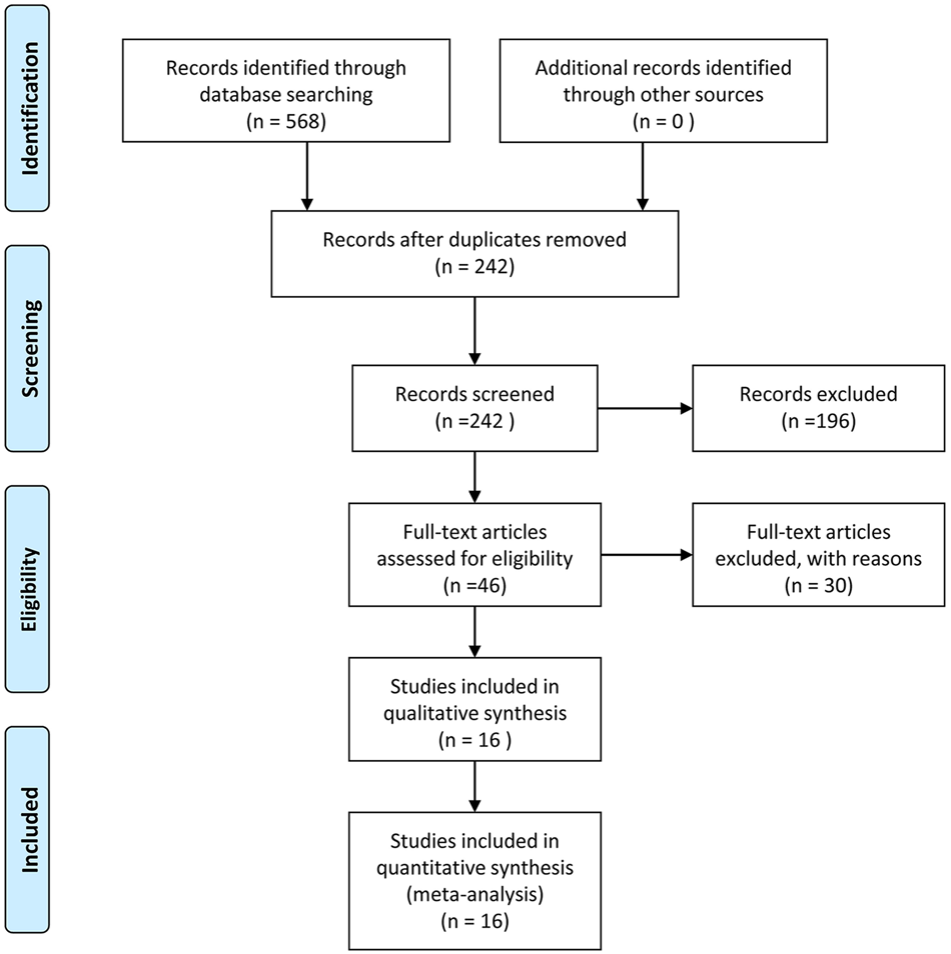
Flowchart of the study selection process

In total, 568 publications were initially identified by searching the electronic database. Among the initial 568 candidate articles, 326 duplicates were removed. Following the first‐round reviewing of titles and abstracts, 46 articles were eligible for second‐round evaluation of full‐text. Finally, 16 publications met predetermined eligibility criteria and were included in this meta‐analysis. **Figure** **1** revealed more details about the search process.

### 3.2 Study characteristics

A total of 16 articles (Brigui et al., 2014; Engelhardt et al., 2015; Gao et al., 2017; Güresir et al., 2011; Hall et al., 2017; Jy et al., 2006; Kh et al., 2010; Liu et al., 2020; Lq & Qx, 2020; Patel et al., 2014; Pr et al., 2006; Sa et al., 2013; Signorelli et al., 2020; Skd et al., 2018; Tan et al., 2015; Yanaka et al., 2003 ) are included in the study, hailing from the United States, South Korea, the United Kingdom, France, Germany, Korea, China, Japan, Britain, and Singapore. The 16 studies with a total of 687 patients that underwent clipping or coiling and reported the complete ONP recovery as the primary outcome were included in this pooled analysis. Of these 687 patients with ONP induced by PcomAA, there were 279 patients from the surgical clipping group versus 408 patients from the endovascular coiling group. At the same time, all 16 articles were single‐center, retrospective studies. More details are shown in **Table** **1**.

**TABLE 1:** Characteristics of publication year, country, study type, cases, and gender in each group for included studies

			Sample Size (*n*)		Gender (F/M)		Age (mean ± standard)		
Author	Country	Type of study	Clip	Coil	Male	Female	Clip	Coil	NOS
**Ahn et al. 2016**	South Korea	Retrospective	7	10	0	17	52.6 years	52.6 years	7
**Brigui et al. 2014**	France	Retrospective	7	13	5	15	60.7 years	49 years	8
**Chen et al. 2006**	USA	Retrospective	7	6	1	12	53.8 years	57.3 years	7
**Engelhardt et al. 2015**	France	Retrospective	9	14	4	19	49.5 years	46.6 years	8
**Guresir et al. 2011**	Germany	Retrospective	4	7	ND	ND	ND	ND	6
**Khan et al. 2013**	USA	Retrospective	8	9	1	16	48.14 years	55.87 years	8
**Nam et al. 2010**	Korea	Retrospective	8	6	1	13	ND	ND	7
**Patel et al. 2013**	UK	Retrospective	9	9	3	15	52.3 years	67.6 years	7
**Tan et al. 2015**	China	Retrospective	132	43	80	95	51.73 years	54.65 years	8
**Yanaka et al. 2003**	Japan	Retrospective	11	1	1	11	60.4 years	60.4 years	5
**Tian et al. 2020**	China	Retrospective	31	39	35	35	60	75	8
**Gao et al. 2017**	China	Retrospective	23	29	8	44	53.9 ± 11.5	54.1 ± 10.0	7
**Hall et al. 2017**	Britain	Retrospective	93	86	29	124	54.1 (23–86)	58.0 (25–82)	6
**Liu et al. 2020**	China	Retrospective	40	112	82	70	55.6±7.9	56.2±8.2	7
**Signorelli et al. 2020**	France	Retrospective	24	31	22	33	ND	ND	7
**Mak et al. 2018**	Singapore	Retrospective	11	11	16	6	57.9	59.6	8

ND = not discussed; F = female; M = male; NOS = Newcastle–Ottawa scale.

### 3.3 Methodological quality

The methodological quality of included studies was evaluated independently by two authors through the Newcastle–Ottawa Quality Assessment Scale (NOS) for nonrandomized controlled trials (non‐RCTs). Briefly, a maximum of 9 points was assigned to each study: 4 for selection, 2 for comparability, and 3 for outcomes. Studies with scores of 7 or more were defined as high methodological quality. Those with scores of 6 or less were defined as poor quality.

Meanwhile, we also used a specific quality aspect to assess the studies such as control of confounding factors according to six criteria, including the items of age, cases with complete initial ONP, the period between ONP onset and therapy, patients with ruptured aneurysm before treatment, size of the aneurysm, and follow‐up period. Studies with scores of 5 or more of control of confounding factors were defined as good. Those with scores of 3 to 4 were defined as an adequate control. The specific score for each study was shown in **Table** **2**.

**TABLE 2:** The literature quality assessment

		Newcastle–Ottawa scale (NOS)				
Author, year	Design	Selection	Comparability	Exposure	Total scores	Control of confounding factors
**Ahn et al. 2016**	Non‐RCT	3	1	3	7	Adequate (4/6)
**Brigui et al. 2014**	Non‐RCT	4	2	2	8	Good (5/6)
**Chen et al. 2006**	Non‐RCT	3	2	2	7	Adequate (4/6)
**Engelhardt et al. 2015**	Non‐RCT	4	2	2	8	Good (6/6)
**Guresir et al. 2011**	Non‐RCT	3	1	2	6	Adequate (3/6)
**Khan et al. 2013**	Non‐RCT	3	2	3	8	Good (6/6)
**Nam et al. 2010**	Non‐RCT	3	1	3	7	Good (5/6)
**Patel et al. 2013**	Non‐RCT	3	2	2	7	Good (5/6)
**Tan et al. 2015**	Non‐RCT	3	2	3	8	Good (5/6)
**Yanaka et al. 2003**	Non‐RCT	2	1	2	5	Adequate (3/6)
**Tian et al. 2020**	Non‐RCT	3	2	3	8	Good (5/6)
**Gao et al. 2017**	Non‐RCT	3	2	2	7	Good (5/6)
**Hall et al. 2017**	Non‐RCT	3	1	2	6	Adequate (4/6)
**Liu et al. 2020**	Non‐RCT	4	1	2	7	Good (5/6)
**Signorelli et al. 2020**	Non‐RCT	3	2	2	7	Good (5/6)
**Mak et al. 2018**	Non‐RCT	3	2	3	8	Good (5/6)

NOS = Newcastle–Ottawa scale; RCT = randomized controlled trials.

## 4 OUTCOMES

### 4.1 Complete ONP recovery at follow‐up

**FIGURE 2:**
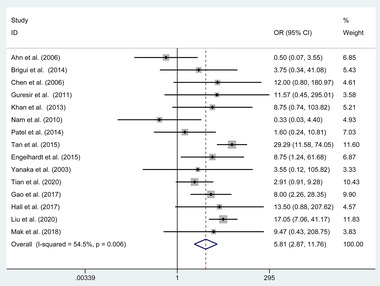
Forest plot of the odds ratio of the complete oculomotor nerve palsy recovery

Complete ONP recovery was reported in 15 of the 16 included studies comprising 632 (91.85%) of 687 patients. 384 (60.76%) of the 632 patients received clipping, of which 248 (39.24%) patients received coiling. The total complete ONP recovery rate was 68.5% (433 of 632 patients) at follow‐up. A random‐effects model was used to assess it owing to the significant heterogeneity (*p* = .006, *I* ^2^ = 54.5%). The pooled evaluation of the overall proportions demonstrated a significantly higher rate of complete ONP recovery that appeared in the clipping group (OR = 5.808, 95%CI 2.87 to 11.76, *p* < .001, **Figure** **2**).

### 4.2 Partial ONP recovery

**FIGURE 3:**
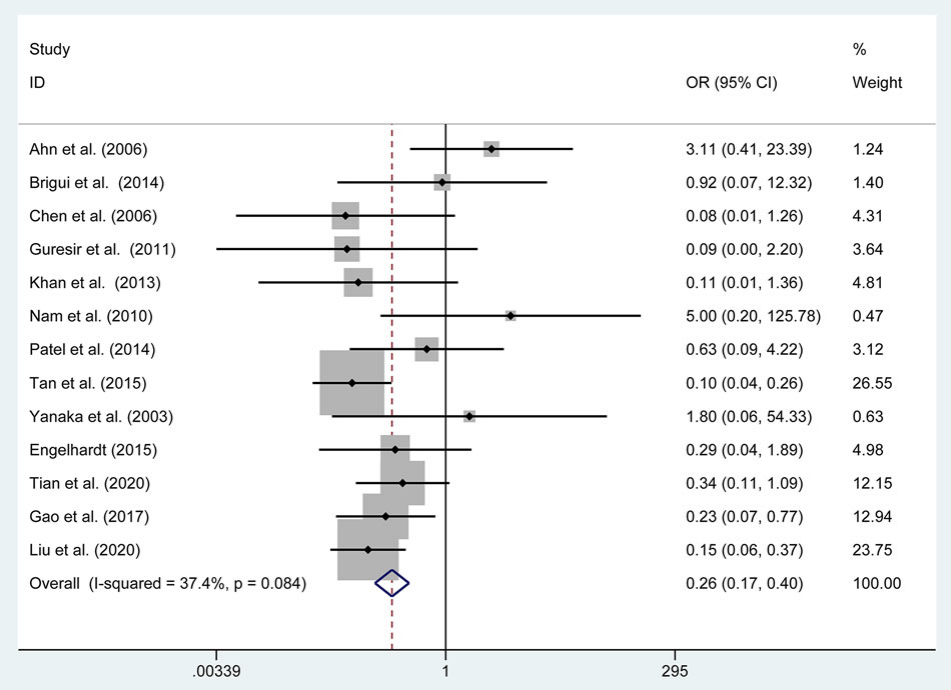
Forest plot of the odds ratio of the partial oculomotor nerve palsy recovery

We adopted meta‐analytical techniques to assess the incidence of postoperative partial ONP recovery. Reviewing the data of the included studies, 13 articles (368 in clipping and 227 in coiling) reported on the partial ONP recovery. We selected the fixed effects model because the heterogeneity was not significantly different (*p* = .084, *I* ^2^ = 37.4%). The coiling group was related to a higher incidence of partial ONP recovery (OR = 0.264, 95% CI 0.173 to 0.402, *p* < .001, **Figure** **3**).

### 4.3 No ONP improvement at follow‐up

**FIGURE 4:**
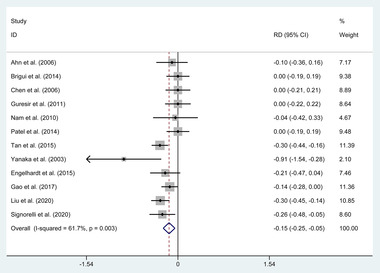
Forest plot of the rate differences of no oculomotor nerve palsy improvement

Twelve publications (358 in endoscopic, 227 in clipping) reported no ONP improvement. A random‐effects model was used to assess it due to the significant heterogeneity (*p* = .003, *I* ^2^ = 61.7%). The pooled evaluation of the overall proportions demonstrated a significantly higher rate of no ONP improvement appeared in the coiling group (RD = −0.149, 95% CI −0.247 to −0.051, *p* = .003, **Figure** **4**).

### 4.4 Postoperative complications

Eight studies reported on complications. No significant heterogeneity was found, then the fixed effects model was selected (*p* = .368, *I* ^2 ^= 8%). We revealed the endoscopic treatment was not related to a lower rate of complications than the clipping group (RD = −0.070, 95% CI −0.146 to 0.006, *p* = .071).

### 4.5 Subgroups of complete ONP recovery based on the condition of patients

#### 4.5.1 Patients with ruptured or unruptured aneurysm

**FIGURE 5:**
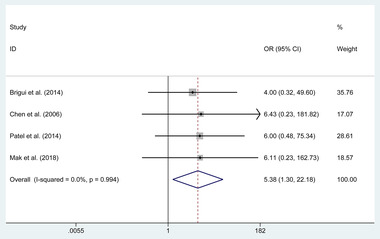
Forest plot of the odds ratio of the complete oculomotor nerve palsy recovery in patients with a ruptured aneurysm

The pooled estimates of the overall proportions showed no significant difference in the incidence of complete ONP recovery in patients with unruptured aneurysm (OR = 0.950, 95% CI 0.506 to 1.783, *p* = .872); however, a significant difference was revealed in the complete ONP recovery in patients with ruptured aneurysm (OR = 5.38, 95% CI 1.304 to 22.181, *p* = .020, **Figure** **5**).

#### 4.5.2 Patients with complete or incomplete initial ONP

**FIGURE 6:**
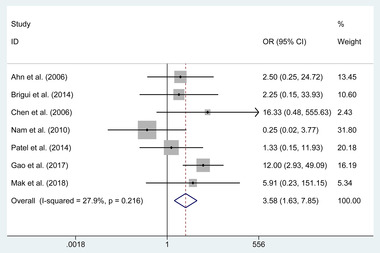
Forest plot of the odds ratio of the complete oculomotor nerve palsy recovery in patients with complete initial ONP

The pooled estimates of the overall proportions showed no significant difference in the incidence of complete ONP recovery in patients with the incomplete initial ONP (RD = 0.266, 95% CI −0.12 to 0.450, *p* = .266); however, a significant difference was revealed in the complete ONP recovery in patients with the complete initial ONP (OR = 3.579, 95% CI 1.632 to 7.851, *p* = .020, **Figure** **6**)

### 4.6 Subgroups of complete ONP recovery based on the quality of studies

#### 4.6.1 Studies with good or nongood control of confounding factors

A significant difference was revealed in the complete ONP recovery in studies with the good (OR = 6.565, *p* < .001) and nongood control of confounding factors (OR = 3.254, *p* = .028)

#### 4.6.2 Studies with good or poor quality based on NOS

A significant difference was revealed in the complete ONP recovery in studies with the good (OR = 5.371, *p* < .001) and poor (OR = 8.730, *p* = .017) quality based on NOS. More details about the results are shown in **Table** **3**.

**TABLE 3:** The postoperative outcomes of this meta‐analysis

		Group's size		Overall effect			Heterogeneity	
Outcomes	Studies numbers	Clipping	Coiling	Effect estimates	95% CIs	*p* Value	*I*^2^ (%)	*p* Value
**Comparison of two interventions for oculomotor nerve palsy induced by posterior communicating artery aneurysms**								
**Complete ONP recovery**	15	384	248	OR (5.808)	2.869 to 11.759	**<.00** **1**	54.5%	.006
**Partial ONP recovery**	13	368	227	OR (0.264)	0.173 to 0.402	**<.001**	37.4%	.084
**Postoperative complications**	8	363	227	RD (−0.070)	–0.146 to 0.006	.071	8%	.368
**No ONP improvement**	12	358	227	RD (−0.149)	–0.247 to −0.051	**.003**	61.7%	.003
**Subgroups of complete ONP recovery based on the condition of patients**								
**Ruptured aneurysm**	4	22	24	OR (5.379)	1.304 to 22.181	**.020**	0.0%	.994
**Unruptured aneurysm**	10	97	112	OR (0.950)	0.506 to 1.783	.872	12.2%	.331
**Complete initial ONP**	7	52	61	OR (3.579)	1.632 to 7.851	**.001**	27.9%	.216
**Incomplete initial ONP**	7	19	23	RD (0.163)	–0.124 to 0.450	.266	0.0%	.901
**Subgroups of complete ONP recovery based on the methodological quality of studies**								
**Good control of factors**	10	350	214	OR (6.565)	2.989 to 14.422	**<.001**	59.3%	.009
**Nongood control of factors**	5	34	34	OR (3.254)	1.137 to 9.318	**.028**	33.7%	.197
**Good quality based on NOS**	12	364	230	OR (5.371)	2.412 to 11.956	**<.001**	63.8%	.001
**Poor quality based on NOS**	3	20	18	OR (8.730)	1.469 to 51.883	**.017**	0.0%	.820

CIs = confidence intervals; RD = rate difference; OR = odds ratio; ONP = oculomotor nerve palsy; NOS = Newcastle–Ottawa scale.

The bold values represent *P*<0.05.

## 5 DISCUSSION

Although ONP is a common clinical complication of PcomAA, the mechanism between PcomAA and ONP remains unknown (Gao et al., 2017). The differences in the incidence of recovery of ONP induced by PcomAA according to various methods of therapy increase the controversy about OPN's pathogenesis. Previously, it seems that the ONP is induced by direct anatomical compression of PcomAA, which can result in a nerve distortion and impairment of circulation of blood vessels (Stiebel‐Kalish et al., 2003). Currently, the aneurysmal pulsations are also regarded as a mechanism for the ONP induced by PcomAA (Hall et al., 2017).

In the wider controversy on the relative their own pros and cons of clipping and coiling treatment for intracranial aneurysms, there was a more violent debate about whether the clipping or coiling was better for treating ONP caused by PcomAA (Zheng et al., 2017). Although the clipping was associated with a larger trauma compared to coiling (Gaberel et al., 2016), it could directly decline compression of aneurysms to ONP, suppress rebleeding of a lesion, promote the recovery of ONP. In addition, theoretically, endovascular intervention could promote the recovery of ONP by reducing the pulsation of the aneurysm. Because the thrombosis formation in the lesion and contraction of the vessel wall could reduce the pulsation (Brigui et al., 2014). In this study, we performed a meta‐analysis to evaluate whether there was an advantage in terms of ONP caused by PcomAA between surgical method and endovascular coiling.

A systematic review conducted by Guresir et al. (2011) gathered a total of 26 studies and analyzed their findings to found the rate of complete recovery of ONP was 55% and 32% in the surgical and coiling groups respectively. Chen et al. (2006) also demonstrated a significant increment in the rate of complete ONP recovery after the surgical (6/7) method compared to endovascular intervention (2/6). First, we also analyzed the rates of recovery of ONP caused by PcomAA after the treatment of clipping and coiling respectively, and it was divided into complete ONP recovery at follow‐up, partial ONP recovery at follow‐up, and no ONP improvement at follow‐up. We demonstrated that the clipping group was related to a higher incidence of complete ONP recovery at follow‐up (OR = 5.808, 95% CI 2.87 to 11.76, *p* < .001), the lower rates of partial ONP recovery at follow‐up (OR = 0.264, 95%CI 0.173 to 0.402, *p* < .001), and no ONP improvement at follow‐up (RD = −0.149, 95% CI −0.247 to −0.051, *p* = .003).

Meanwhile, we conducted the subgroups of complete ONP recovery according to the condition of patients and the quality of studies. In the subgroup of patients with ruptured or unruptured aneurysms, a significant difference was revealed in the rate of complete ONP recovery in patients with ruptured aneurysms (OR = 5.38, *p* = .020). Similarly, in the subgroup of patients with the complete or incomplete initial ONP, the treatment of clipping was associated with a higher incidence of complete ONP recovery in patients with the incomplete initial ONP (OR = 3.579, *p* = .020). Besides that, in the subgroups of studies with good or nongood control of confounding factors, the clipping group also was related to a higher rate of the complete ONP recovery in studies with the good (*p* < .001) and nongood control of confounding factors (*p* = .028). Similar results also appeared in the subgroup of studies with good or poor quality based on NOS (*p* < .05).

In this study, the overall rates of the complete ONP recovery are 85.42% and 42.34% in the clipping and coiling group respectively. With regard to the reason for the higher rates of complete ONP recovery in the clipping group, we have a couple of guesses. First, although the surgical method and coiling could decline blood flow and then reduce the pulsation of the aneurysm, clipping could directly prevent the decompression of the aneurysm to the oculomotor nerve. Meanwhile, an aneurysm can have a temporary expansion after the treatment of endovascular intervention because of the formation of thrombosis in the cavity of the aneurysm, and the temporary expansion of aneurysm could lead to a direct decompression on the oculomotor nerve. Although the expanded aneurysm was not eliminated after the treatment of coiling, the OPN could also be solved in some patients. That is to say, coiling could promote the recovery of OPN mainly by reducing pulsation of the aneurysm, which stemming from aneurysmal shrinkage and fibrosis induced by the formation of thrombosis. Finally, in patients with a ruptured aneurysm, clipping could remove the intracranial hematoma directly and rinse the blood on the surface of the vessel, then prevent the stimulation of blood on the oculomotor nerve, while blood could only be absorbed by itself in patients receiving coiling (Liu et al., 2020).

Several limitations should be noted in this meta‐analysis. First, the present analysis is mainly limited by the poor quality of the retrospective and small studies, which prevents from drawing definitive conclusions. Second, it is important to take into account the heterogeneity of the patient population studied when interpreting the results of a meta‐analysis, but this meta‐analysis is of the high heterogeneity of the statistical results. Finally, the follow‐up of included studies is not the same. Therefore, although many meaningful conclusions could be revealed about the trends in the recovery of ONP, further high‐quality studies are necessary to determine which procedure is optimal for treating ONP caused by PcomAA.

## 6 CONCLUSION

Surgical clipping was more favorable to the recovery from ONP caused by PcomAA endovascular coiling due to a higher rate of recovery and recovery degree of ONP. Besides that, more evidence‐based performance is needed to supplement this opinion.

### PEER REVIEW

The peer review history for this article is available at https://publons.com/publon/10.1002/brb3.2263.

## AVAILABILITY OF DATA AND MATERIALS

The datasets used and/or analyzed during this study are available from the corresponding author upon reasonable request.

## COMPETING INTERESTS

The authors declare that they have no competing interests.
